# Soft, biocompatible materials and skin-like electronics as wearable devices: an interview with John A. Rogers

**DOI:** 10.1093/nsr/nwac191

**Published:** 2022-09-17

**Authors:** By Enming Song

**Affiliations:** Enming Song is a Professor at the Shanghai Frontiers Science Research Base of Intelligent Optoelectronics and Perception, Institute of Optoelectronics, Fudan University

## Abstract

Recent advances in active materials, micro-fabrication techniques, ultra-miniaturized design approaches and hybrid device layouts form the foundations for emerging classes of unusual systems that can capture physiological signals from the human body in a physically imperceptible fashion at nearly any anatomical location, external on the skin or internal on vital organ systems. Of particular interest with these technologies are their thin geometries, flexible/stretchable physical properties and unique form factors, to enable conformal and gentle contacts with soft, curved and dynamic surfaces of living tissues. Commercial embodiments of skin-integrated devices with clinical-grade monitoring capabilities are just now becoming widely available not only in developed countries but also in the most resource-constrained areas of the globe. A specific example is in skin-like—sometimes referred to as ‘epidermal’—wireless electronics for continuous monitoring of essential markers of physiological health status, with accuracy and reliability comparable to that of expensive, wired-based systems currently used with patients in intensive care units, but cost-effectively and applicable in any setting—in hospitals, health clinics, work environments or in the home. These technologies have the potential to revolutionize diagnostic and therapeutic approaches for the care of patients.

*NSR* spoke to one of the scientific leaders in this field—a 2009 MacArthur Fellow, the winner of the 2022 US National Academy of Sciences James Prize in Science and Technology Integration and one of the few individuals in history to be elected to all three US National Academies, namely the National Academy of Engineering, the National Academy of Science and the National Academy of Medicine, Professor John A. Rogers, the Director of the Querrey-Simpson Institute of Bioelectronics at Northwestern University on the recent advancements and the prospects of soft, biocompatible electronic systems and skin-like wearable devices.

## POWERFUL ENGINEERING DESIGN OPPORTUNITIES OF ‘EPIDERMAL ELECTRONICS’

***NSR*:** What are epidermal electronic systems? What is the motivation for research on these technologies and why are these classes of devices important?

***Rogers*:** The broader motivation is to find ways to exploit the types of function available in Man's most sophisticated forms of electronics—the silicon integrated circuit—to monitor, study and modulate natural processes of the human body, including those of the brain, the most sophisticated form of electronics in the biological world. The challenge is not only in developing biocompatible materials, but also in creating classes of electronics that have soft mechanical properties and curved shapes matched to those of soft living tissues. The goal is to develop materials and design approaches and manufacturing schemes for devices that blur the distinction between biology and technology, in ways that lead to important consequences for biomedical research and clinical healthcare. The required interdisciplinary advances span many traditional areas of study in science, engineering and medicine. We, and others working in the field, believe that these technologies will help to improve human health and to enhance our understanding of living systems.

Applications of such technologies are most straightforward in the context of the skin, due to the minimally invasive interfaces and negligible health risks associated with skin-mounted devices compared to those that are being explored, in parallel, as advanced implantable systems. The ideal skin-interfaced technology is one that mimics all of the key physical properties of the skin itself—its thickness, thermal mass, water/water vapor permeability, weight per unit area and elastic modulus. Devices of this type can laminate gently on the surface of the skin, as a ‘second skin’, with negligible perturbation to natural motions and processes, in a manner that allows mounting at nearly any body location, in a physically imperceptible mode. When engineered with relevant materials, the persistent physical contact between ‘epidermal’ electronics and the skin enables measurements of underlying physiological processes associated with health status, currently only accessible using sophisticated external instruments and wired interconnections, exclusive to hospital or laboratory settings. An overall goal is to embed into this epidermal electronic framework multiple types of sophisticated sensor technologies, wireless data communication capabilities, sources of power supply and so on.

**Figure:**
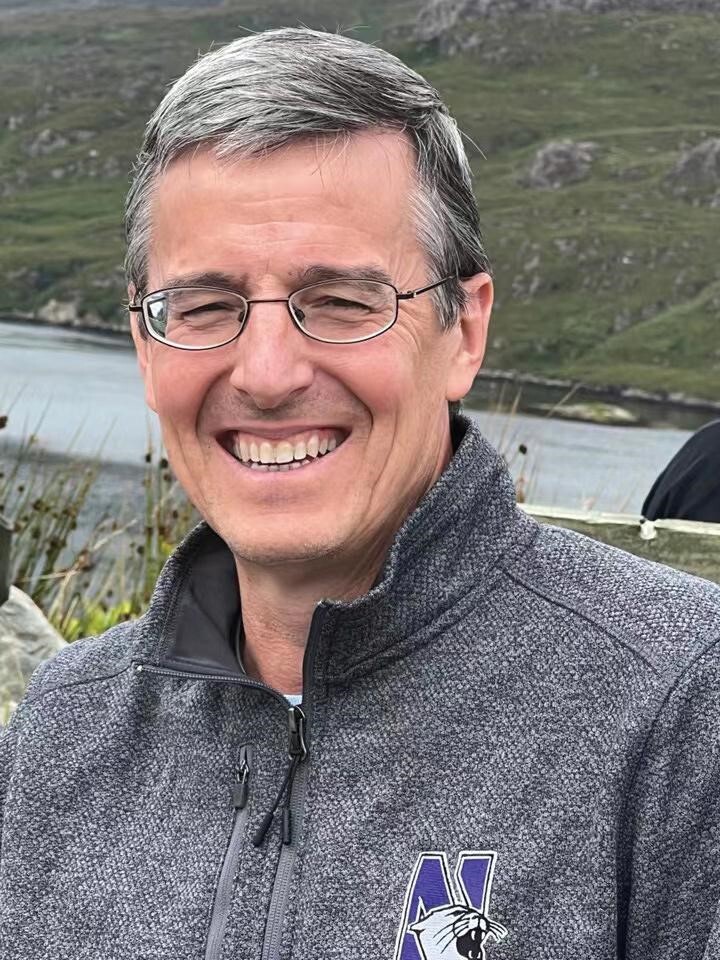
John Rogers is one of the few individuals in history to be elected to all three US National Academies (*courtesy of Prof. John Rogers*).

An overall goal is to embed into this epidermal electronic framework multiple types of sophisticated sensor technologies, wireless data communication capabilities, sources of power supply and so on. —John Rogers

***NSR*:** What strategies in engineering science offer promise in realizing such systems? What are the main difficulties?

***Rogers*:** From an engineering standpoint, it is useful to take a step back and ask the question: What are the key engineering design principles that underpin function in biological systems, and is it possible to adapt certain of these for use in man-made devices? Skin is a remarkably complex organ, and the largest organ of the human body. The functional elements span a wide range of length scales, and they involve heterogeneously integrated, 3D configurations with fluidic, chemical, thermal, mechanical and electrical aspects supported by materials with mechanical properties that range from very soft to relatively stiff. Our strategy has been to pursue engineering approaches that are likewise hybrid, hierarchical and multifunctional in their construction—combining hard and soft materials together in architectures that afford skin-like properties but also incorporate semiconductor device functions enabled by manufacturing flows that can leverage those used in the integrated circuit industry.

The essential ideas revolve around two simple concepts in physical mechanics. The first is that nanoscale structures—nanowires, nanoribbons, nanomembranes, etc.—have exceptionally low flexural rigidities even with constituent materials that have high elastic moduli, like most inorganic semiconductors. These floppy, flexible elements can serve as building blocks for high-performance electronics formed on thin, bendable substrates. Integration with soft tissues like the skin, however, requires technologies that not only bend but also stretch, sometimes to levels of strain that can reach tens of percents. The second simple concept addresses this challenge by exploiting controlled mechanical buckling—a phenomenon traditionally viewed by the engineering community as a failure mechanism to be avoided. Specifically, we bond collections of nanomaterial structures like silicon nanoribbons to an underlying soft, elastomeric support in a state of pre-strain. Relaxing the strain spontaneously transforms the initially flat nanoribbons into ‘wavy’ geometries, thereby creating a hard–soft material composite in which the elastomer defines the overall mechanical properties and the silicon supports the electronic functionality. The wavelengths and amplitudes of these wavy structures change and automatically adapt as externally imposed forces stretch, compress, twist or even tie the system into knots, in a way that avoids fracture-inducing strains in the silicon. Elaborations and extensions of these two basic concepts yield practical pathways to nearly any class of electronics, in physical forms that can, through careful mechanics modeling and choices in materials and design layouts, precisely match the characteristics of a targeted tissue, such as the epidermis.

Many researchers have made contributions in this field of research over the years since the introduction of these ideas. Most notably, the group of Professor Yonggang Huang, a colleague and long-time close collaborator here at Northwestern University, has pioneered analytical and computational techniques for quantifying the mechanics of these systems. The resulting numerical tools can be used to guide design choices for layouts, fulfilling a role analogous to that of circuit simulators but for materials and mechanics rather than devices and electronics.

***NSR*:** What is the current state of the art in this area? What are the main areas of future opportunity?

***Rogers*:** The broader community of researchers in this area has demonstrated a wide range of sensors that are compatible with this epidermal platform—from those that precisely measure various types of bio-potentials, to those that characterize thermal behaviors, mechanical strains, physical movements, optical properties, mechano-acoustic phenomena and many others. Some yield data that quantitatively align with those generated by traditional methods used in clinical practice; others represent novel metrics of health. A single device can support multiple sensors and multiple devices can be located at different regions of the body, for multi-modal and time-synchronized multi-nodal operation. Specific use cases range from patients with cardiopulmonary disorders to those with aphasia or dysphagia to individuals who suffer from hydrocephalus, and to a broad diversity of others, spanning the entire age spectrum—from neonates to the elderly. Prototypes and early commercial platforms are now deployed not only in the hospital but also in the home, during natural daily activities.

Ongoing and future work seeks to build on this progress and also to aggressively extend measurement capabilities to biochemical assessments of health, through sensors that can track biomarkers non-invasively via these skin interfaces. The emergence and growing widespread use of continuous glucose monitors, as an example, may provide hints of the sort of progress that might be possible in epidermal electronic sensors for other important chemical and biochemical species.

Ongoing and future work seeks to build on this progress and also to aggressively extend measurement capabilities to biochemical assessments of health, through sensors that can track biomarkers non-invasively via these skin interfaces.

—John Rogers

## NEW APPLICATIONS AND CHALLENGES FOR WEARABLE ELECTRONIC DEVICES

***NSR*:** Have any of these technologies been translated into practical applications? What are they? Who might benefit from them?

***Rogers*:** Some of the earliest commercial examples of epidermal electronics include the My UV Patch, a product that we produced with L’Oreal as a consumer, single-use device for tracking sun exposure, and the Biostamp, a medical monitor commercialized by MC10 (since acquired by Medidata) for capturing physiological data in support of human clinical trials, with approvals from the US Food and Drug Administration (FDA). The most advanced technologies now form the basis for a line of FDA-approved products offered by Sibel Health, in funded partnerships with large companies such as Drager, General Electric, SpaceLabs and Elevance for applications spanning hospital care to remote patient monitoring. Separate efforts at the company Rhaeos focus on a flow-sensing platform for monitoring the function of shunts used with hydrocephalus patients, currently in the final stages of pivotal trials across multiple children's hospitals here in the USA, with breakthrough designation by the FDA.

For epidermal devices that include microfluidic capabilities, the highest-volume product monitors sweat loss, sweat rate and electrolyte loss as information to guide precision management of hydration status in sports and athletic performance. Here, 3 million devices have been delivered or are in various stages of high-volume production by the company Epicore Biosystems, as the Gx Patch, launched in a partnership with Gatorade. Extended versions of this technology are in advanced stages of commercialization for nutrition/wellness and for worker safety, the latter geared initially toward the oil/gas industry through a funded collaboration with Chevron.

We, and other groups in independent efforts, are working on additional devices that are entering the translational pipeline, all of which exploit the same core ideas in soft, skin-compatible wireless electronics—functional near-infrared spectrometry systems, multi-lead devices for recording electroencephalograms and fetal electrocardiograms, multi-nodal collections of sensors for detecting early signs of neurodevelopmental disorders and devices to track swallowing behaviors in patients with Alzheimer's dementia and Parkinson's disease. These advanced development efforts are supported by federal funding agencies and philanthropic organizations including BMGF (the Bill and Melinda Gates Foundation), the Ryan Family Foundation and the Michael J. Fox Foundation. The most significant philanthropy that supports the academic research that underpins all of these technologies is from Kimberly Querrey and Louis Simpson, through their support of the Querrey-Simpson Institute for Bioelectronics here at Northwestern.

***NSR*:** What are the most compelling application opportunities from your point of view?

***Rogers*:** Some of the most important and immediate opportunities are in monitoring of high-risk, vulnerable patients, where our primary focus is on maternal, fetal, neonatal and pediatric health. In fact, our first targeted effort was in care for premature babies—extremely precious but tiny, fragile patients, with highly uncertain and rapidly variable health status. In neonatal intensive care units (NICUs) these patients are strictly monitored, across all vital signs, continuously and with clinical-grade precision. The clinical-standard hardware for these purposes relies on hard biosensors affixed to the skin with strong adhesive tapes and interfaced to expensive external data-acquisition systems through hard-wired connections. Application and removal of these sensors can cause injury to the delicate neonatal skin. The wired interfaces disrupt natural movements of the babies, they complicate even the most routine aspects of clinical care and they frustrate therapeutic skin-to-skin contact and physical engagement with parents and health workers. The idea, pursued in a broad, collaborative team of medical professionals and academic engineers starting in the year of 2016, was to replace that technology with wireless epidermal devices to eliminate these practical limitations and also to establish a more cost-effective and more readily deployable means for monitoring. As published in 2019, two time-synchronized wireless epidermal devices can reproduce the entire set of essential vital signs measurements that represent the standard of care in the most advanced NICUs, not only with clinical-grade accuracy but also with improved reliability and reduced measurement artifacts.

**Figure:**
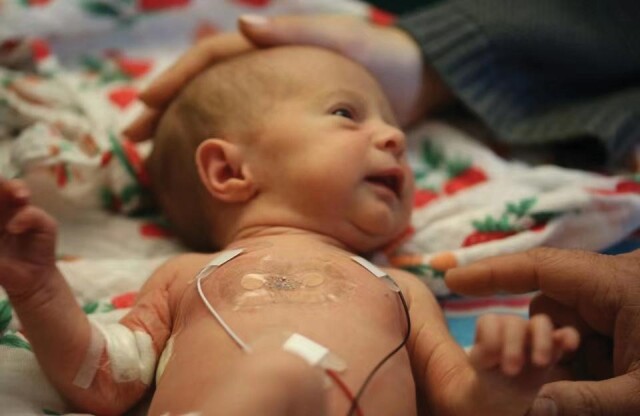
A wireless, epidermal device mounted on the neonate's chest, monitoring its health status (*credit: Rogers Research Group*).

We have now deployed these technologies onto hundreds of neonates in operating NICUs across hospitals here in the Chicago land area, and we are completing a demonstration of the world's first fully wireless NICU room at a hospital in Montreal and another in Antwerp. We have also migrated these technologies beyond the NICU and into the pediatric intensive care unit, where they add similar value. With adapted and extended versions of these platforms, we can also monitor maternal and fetal health throughout the intrapartum period. In partnerships with BMGF and the Save the Children (STC) organization, we have deployed thousands of these devices into India, Pakistan, Zambia, Kenya and Ghana. About 10 000 babies and expecting mothers have been evaluated, as part of ongoing studies and clinical trials.

***NSR*:** What about applications in syndromic tracking of infectious diseases, like the COVID-19 pandemic?

***Rogers*:** In late March of the year of 2020, we were contacted by members of the medical community in Chicago about the possibility of adapting a device that we developed to monitor swallowing and speaking behaviors in stroke survivors to enable continuous measurements of key symptoms associated with COVID-19. In response, we spent the next several weeks rapidly building out a complete, customized system—hardware, firmware, software and cloud data interfaces—for monitoring cough, respiratory behavior, heart rate, heart rate variability and core body temperature in an automated fashion with minimal user burden. We produced hundreds of these devices using low-volume manufacturing tools in our labs and we deployed them across frontline healthcare workers at the Shirley Ryan AbilityLab (a rehabilitation hospital) and at Northwestern Memorial Hospital, and onto Covid patients both during their stay in these clinical settings and for some weeks after their release into the home. To support these programs, we were designated as essential workers with full lab access throughout the lockdown period. Thousands of individuals were monitored, not only here in Chicago, supported by competitive grants from the Biomedical Advanced Research and Development Agency and the National Science Foundation, but also in India through support from the Indian government and in Kenya and Ghana with the BMGF and the STC organization.

We feel that there is strong potential for using these types of technologies for monitoring the health status not only of individuals but also of populations, as a mechanism for tracking the spread and progression of diseases, far beyond possibilities afforded by more primitive types of measurements that are possible with conventional wearable devices. An important additional goal is in early detection of the disease and as a digital diagnostic that could complement more traditional molecular tests.

## ONGOING EFFORTS AND FUTURE DIRECTIONS

***NSR*:** Can we adapt the foundational ideas in epidermal electronics for other applications? Where do you see the future of such technologies going?

***Rogers*:** We see two near-term opportunities for taking the basic engineering concepts that enable epidermal sensors and applying them to other areas of interest. The first is in using the skin not as the basis for a sensing interface but as a sensory input channel, through haptic engagement with mechanoreceptors in the skin. Of course, haptic technologies have a long history, starting with vibrational motors integrated into systems that sit on desktops, as haptic inputs to the hand at the point of contact. Later, such approaches began to appear in portable devices, ranging from power tools, to game controllers, to smartphones and smart watches. The most recent trend is in wearable interfaces, dominated by gloves that engage haptic actuators at the fingertips, as the basis for adding skin sensations to video and audio inputs in virtual and augmented reality experiences, perhaps of essential importance in the emerging metaverse. Epidermal electronic devices, when instrumented with haptic actuators, offer the potential for large-area, distributed engagement across all regions of the body, beyond just the fingertips. Our work demonstrates that such interfaces—soft, flexible sheets with arrays of independently controlled haptic actuators—are possible, with real-time wireless internet connectivity and capabilities for producing different skin sensations with minimal latency, including those that roughly resemble touching, stroking, pinching, twisting and squeezing. Our main interest is in interfaces that provide feedback of medical relevance, where an early example is in devices that assist stroke survivors and patients suffering from dementia to control their swallowing and speaking behaviors. Mass market consumer applications are in entertainment, gaming and remote social interactions.

The second opportunity is in thinking beyond electronic and haptic modes of operation to skin-mounted lab-on-a-chip technologies—analytical chemistry laboratories on the skin. Here, epidermal systems incorporate networks of microchannels, valves, reservoirs and biochemical sensors for capturing,

The same ideas that can serve as the basis for devices that interface with the skin can be exploited for those that monitor internal organs—the heart, brain, bladder, peripheral nerves, spinal cord, lungs, diaphragm and others are in active development.

—John Rogers

storing and analysing biomarkers in pristine, microliter volumes of sweat as it emerges from the surface of the skin. At the simplest level, such devices can quantify sweat rate and sweat loss, along with electrolyte loss, as quantitative information to ensure healthy management of hydration levels. Other demonstrated possibilities range from tracking blood alcohol through analysis of sweat alcohol, to monitoring exposures to toxic or banned substances, to evaluating loss channels for essential amino acids. These microfluidic systems can also integrate epidermal electronics for electrochemical sensing methods, or they can exploit colorimetric chemical reagents as a cost-effective alternative.

***NSR*:** Can related approaches be used for implanted devices? Are there other opportunities in this area of bio-integrated technologies?

***Rogers*:** Yes, the same ideas that can serve as the basis for devices that interface with the skin can be exploited for those that monitor internal organs—the heart, brain, bladder, peripheral nerves, spinal cord, lungs, diaphragm and others are in active development. Additional options follow from the use of biodegradable materials, to form devices that operate in the body for a time period aligned with a natural biological process like wound healing and then disappear naturally to avoid the need for a surgical extraction. Representative examples include electrical stimulators for damaged nerves and for temporary cardiac pacing, vehicles for wirelessly programmed drug delivery and sensors for intracranial pressure. Exploratory directions that involve 3D architectures are also interesting, as the basis for volumetric levels of tissue integration, with initial demonstrations in bioelectronic interfaces to spheroids, organoids and assembloids. An incredible range of opportunities, with aspects that cut across nearly every area of physical and biological research—of both basic and applied scientific interest, with potential to improve every aspect of human healthcare.

